# Premature senescence of the liver in Alagille patients

**DOI:** 10.1371/journal.pone.0285019

**Published:** 2023-04-26

**Authors:** Giulia Jannone, Catherine de Magnée, Roberto Tambucci, Jonathan Evraerts, Joachim Ravau, Mustapha Najimi, Etienne Marc Sokal

**Affiliations:** 1 Laboratory of Pediatric Hepatology and Cell Therapy, Institut de Recherche Expérimentale et Clinique (IREC), UCLouvain, Brussels, Belgium; 2 Pediatric Surgery and Transplantation Unit, Department of Surgery, Cliniques Universitaires Saint-Luc, UCLouvain, Brussels, Belgium; University of Minnesota Medical School, UNITED STATES

## Abstract

**Introduction:**

Alagille syndrome (ALGS) is an autosomal dominant disease characterized by a multisystem involvement including bile duct paucity and cholestasis, caused by *JAG1* or *NOTCH2* mutations in most of the cases. Jagged1-Notch2 interactions are known to be crucial for intrahepatic biliary tract development, but the Notch signaling pathway is also involved in the juxtacrine transmission of senescence and in the induction and modulation of the senescence-associated secretory phenotype (SASP).

**Aim:**

Our aim was to investigate premature senescence and SASP in ALGS livers.

**Methods:**

Liver tissue from ALGS patients was prospectively obtained at the time of liver transplantation (n = 5) and compared to control livers (n = 5).

**Results:**

We evidenced advanced premature senescence in the livers of five *JAG1* mutated ALGS pediatric patients through increased senescence-associated beta-galactosidase activity (p<0.05), increased p16 and p21 gene expression (p<0.01), and increased p16 and γH2AX protein expression (p<0.01). Senescence was located in hepatocytes of the whole liver parenchyma as well as in remaining bile ducts. The classical SASP markers TGF-β1, IL-6, and IL-8 were not overexpressed in the livers of our patients.

**Conclusions:**

We demonstrate for the first time that ALGS livers display important premature senescence despite Jagged1 mutation, underlying the complexity of senescence and SASP development pathways.

## Introduction

Alagille syndrome (ALGS) is an autosomal dominant disease characterized by a multisystem involvement including bile duct paucity and cholestasis. The penetrance is highly variable and clinical manifestations can also consist in cardiovascular defects, renal anomalies, characteristic facial features, vertebral arch/other skeletal defects and ocular features (typically posterior embryotoxon) [1]. In the presence of ALGS-related initial cholestasis, more than 75% of the patients need a liver transplantation before the age of 18 years [2]. A mutation in *JAG1*, which encodes the protein Jagged1, is associated with the phenotype in more than 90% of the cases. *NOTCH2* mutations are found in up to 4% of the patients in the absence of *JAG1* anomalies [2, 3]. Jagged1-Notch2 interactions are known to be crucial for intrahepatic biliary tract development [4]. The Notch signaling pathway also demonstrated to have major effects in senescence induction and propagation, especially through Notch1 [5]. Jagged1-Notch1 interactions induce a juxtacrine transmission of senescence and Notch1 mediates a switch between TGF-β-rich secretome and pro-inflammatory secretome during senescence [6]. Activation of Notch1 or Notch2 also induced premature senescence *in vitro* in human primary endothelial cells [7]. It was therefore relevant to investigate the presence of premature senescence and senescence-associated secretory phenotype (SASP) in ALGS livers.

## Materials and methods

### Liver samples

Five patients with genetically confirmed ALGS who underwent liver transplantation were prospectively recruited in the Pediatric Gastroenterology and Hepatology Unit of Cliniques Universitaires Saint-Luc between 2018 and 2022. A fragment of the explanted liver was collected during the surgery and biochemical data were obtained the day before the procedure. Five control liver fragments were provided by the Cliniques Universitaires Saint-Luc biobank when consent for research purposes was given. One control liver fragment was obtained from an explanted liver during liver transplantation for hyperoxaluria type 1 (metabolic defect in a structurally healthy liver), while the four others were obtained from diseased donors when the liver was not approved for liver transplantation (e.g. partially damaged organ) or when part of the liver was not transplanted due to surgical considerations. Biochemical data was not available for control patients. This project was approved by the Ethics Committee of Cliniques Universitaires Saint-Luc (registration number B403201938739). Written informed consent was obtained for all study participants.

### Senescence-associated β-galactosidase activity assay

Senescence-associated β-galactosidase (SA-β-gal) activity assay was performed on cryopreserved liver tissue at pH 4 for one hour (37C) as previously described [8]. Stained sections were washed with PBS before performing immunohistochemical staining when indicated.

### Immunohistochemistry

Five μm formalin-fixed paraffin-embedded (FFPE) liver sections were deparaffinized and rehydrated in xylene and graded alcohol series. After a 1 hour blocking step in 5% BSA (Merck, Darmstadt, Germany) ± 5% normal goat serum (Thermo Fisher Scientific, Waltham, MA, USA), sections were incubated at 37C with primary antibody for 1 hour (S1 Table in S1 File) followed by species-specific secondary antibodies incubation (Dako EnVision+ System HRP; Agilent, Santa Clara, CA, USA) and 3,3′-diaminobenzidine (DAB) chromogen detection. Digital quantification of staining was assessed on x20 magnification scanned sections by using the image analysis tool Author version 2017.2 (Visiopharm, Hørsholm, Denmark) as previously described [8].

### Reverse transcription quantitative polymerase chain reaction

Liver homogenates were obtained from liver biopsies with the FastPrep-24 Classic Instrument (MP Biomedicals, Irvine, CA, USA). Total RNA was extracted from liver homogenates using Tripure isolation reagent (Roche, Basel, Switzerland) and retro-transcribed using the high-capacity cDNA reverse transcription kit (Applied Biosystems, Waltham, CA, USA). Quantitative PCR was carried out in duplicate using TaqMan universal MasterMix (Applied Biosystems) and pre-designed TaqMan probes (S2 Table in S1 File). Relative gene expression was determined with the ΔΔCt method using *TBP* and *PPIA* as housekeeping genes [9].

### Statistical analysis

Statistical analysis was conducted using GraphPad Prism 5.0 (GraphPad Software, La Jolla, CA, USA). Continuous variables were presented as mean ± standard deviation (SD). The non-parametric Mann-Whitney U test was used to compare continuous variables between subgroups. A two-tailed p-value < 0.05 was considered to indicate statistical significance for all analysis.

## Results

**Fig 1:**
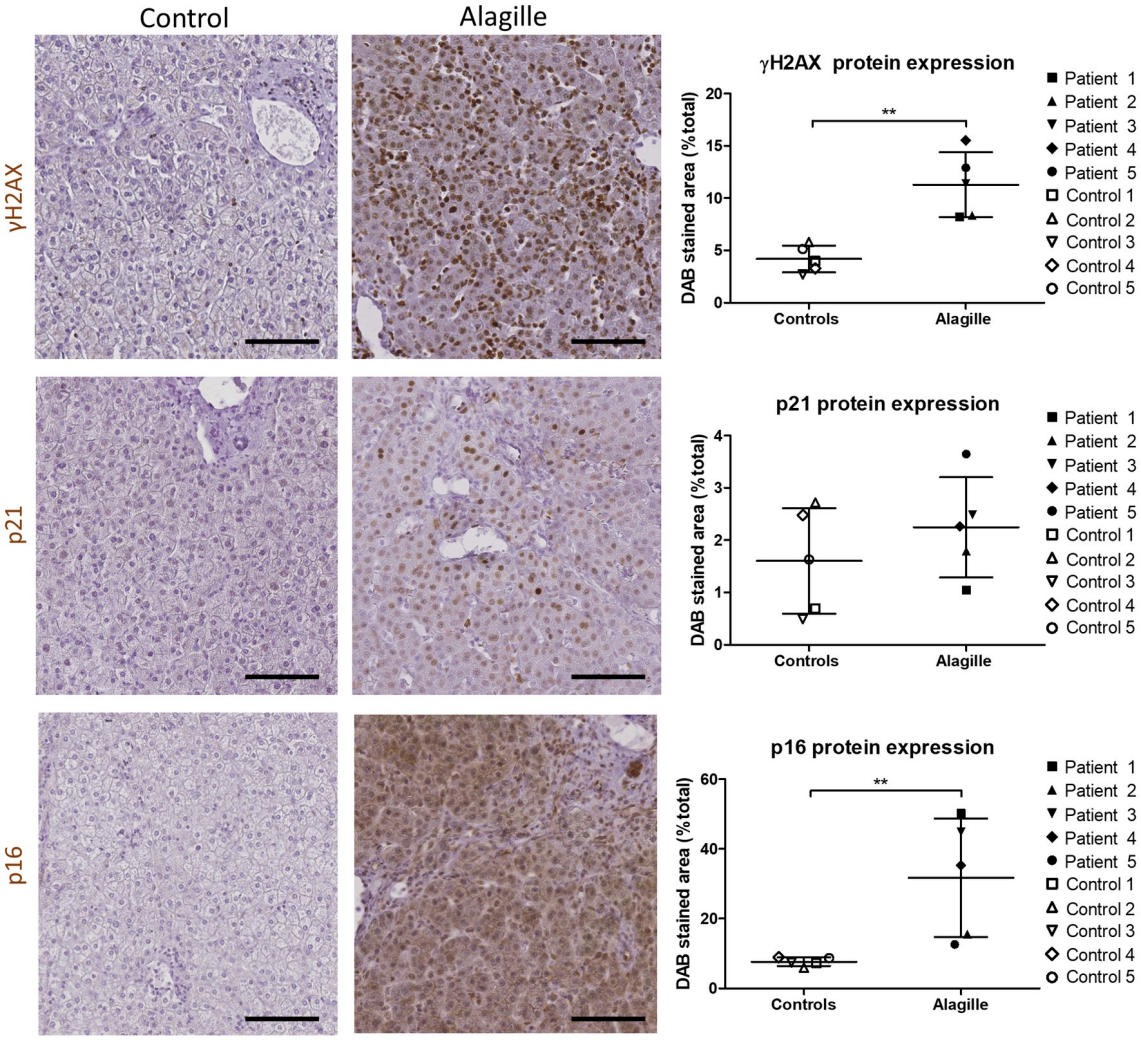
Protein expression of senescence markers in ALGS livers. Protein expression of γH2AX and p16 increases in ALGS livers as compared to controls. No significant change is observed regarding p21 protein expression. Liver sections from patient number 1 and control number 3 were used as representative images. ALGS: Alagille syndrome; DAB: 3,3′-diaminobenzidine. Data is presented as mean ± SD; **p<0.01. Scale bars = 100 μm.

**Fig 2:**
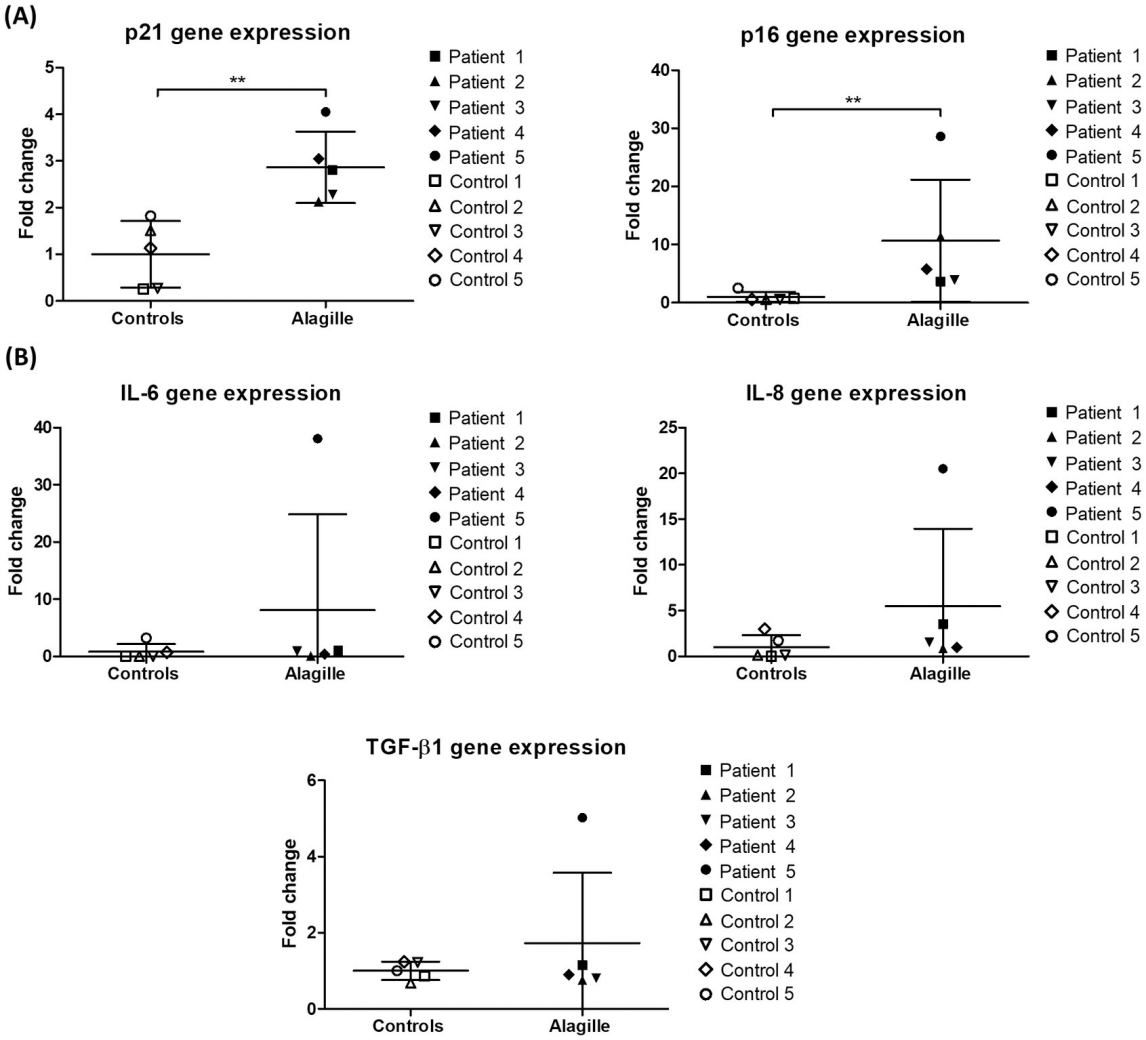
Gene expression of senescence and SASP markers in ALGS livers. (A) Gene expression of p21 and p16 increases in ALGS livers. (B) Gene expression of SASP markers TGF-β1, IL-6 and IL-8 is unchanged in ALGS livers. ALGS: Alagille syndrome; SASP: senescence-associated secretory phenotype. Data is presented as mean ± SD; **p<0.01.

Description of the study population is presented in Table 1. All the ALGS patients were transplanted at a pediatric age, while 2/5 controls were adults. All control liver tissues were structurally normal (no histological inflammation nor fibrosis). A heterozygous mutation of *JAG1* was confirmed in all ALGS patients. Two patients were transplanted for end-stage liver disease with cirrhosis and portal hypertension, and three patients were transplanted due to poor quality of life mainly related to pruritus and jaundice, in the absence of established cirrhosis (Metavir F2-F3). Advanced premature senescence was evidenced in ALGS livers as demonstrated by an increase in p16 and γH2AX protein expression, as well as an increase in p16 and p21 gene expression (Figs 1 and 2A). However, gene expression of the SASP-associated markers TGF-β1, IL-6 and IL-8 was not increased in ALGS livers of our cohort (Fig 2B). One ALGS patient (patient number 5) had markedly increased liver gene expression of those three SASP markers, but we could not evidence any relevant clinical, biochemical or histological differences in this patient as compared to the four others except for the presence of hypoalbuminemia and marked hyperbilirubinemia (Table 1).

**Table 1:** Description of the study population.

	Alagille syndrome				
	Patient 1	Patient 2	Patient 3	Patient 4	Patient 5
Age (years)	1	11	4	5	5
Gender	F	F	M	F	M
AST (UI/L) (< 80)	196	321	326	325	757
ALT (UI/L) (< 35)	153	381	394	235	242
γGT (UI/L) (< 40)	335	460	692	131	133
Total bilirubin (mg/dL) (< 1.2)	14.8	1.4	15.6	25.4	36.3
Albumin (g/L) (>34)	38	43	45	38	29
INR (0.8–1.2)	1.01	1.01	1.12	1.4	1.58
Histological fibrosis (Metavir)	F2	F2	F3	F4	F4
Liver transplant indication	Quality of life	Quality of life	Quality of life	End-stage liver disease	End-stage liver disease
*JAG1* mutation	c.551G>A / p.(Arg184His)	c.550C>T / p.(Arg184Cys)	c.2122_2125del, p.(Gln708Valfs*34)	c.2455A>G (p.Ile819Val)	c.886+1G>A
Other anomalies:					
Heart	No	Yes	Yes	Yes	Yes
Kidneys	No	No	Yes	Yes	Yes
Vertebral	No	No	Yes	Yes	Yes
Ophtalmic	No	Yes	Yes	No	No
Typical facies	Yes	Yes	Yes	Yes	Yes
	**Controls**				
	**Control 1**	**Control 2**	**Control 3**	**Control 4**	**Control 5**
Age (years)	4	28	0	15	41
Gender	M	F	M	M	F
Histological fibrosis (Metavir)	F0	F0	F0	F0	F0
Liver transplant indication	/	/	/	Hyperoxaluria type 1	/
Cause of death	Status epilepticus	Motor vehicle accident	Neonatal asphyxia	/	Hemorragic stroke

Standard values are indicated for all biochemical parameters.

**Fig 3:**
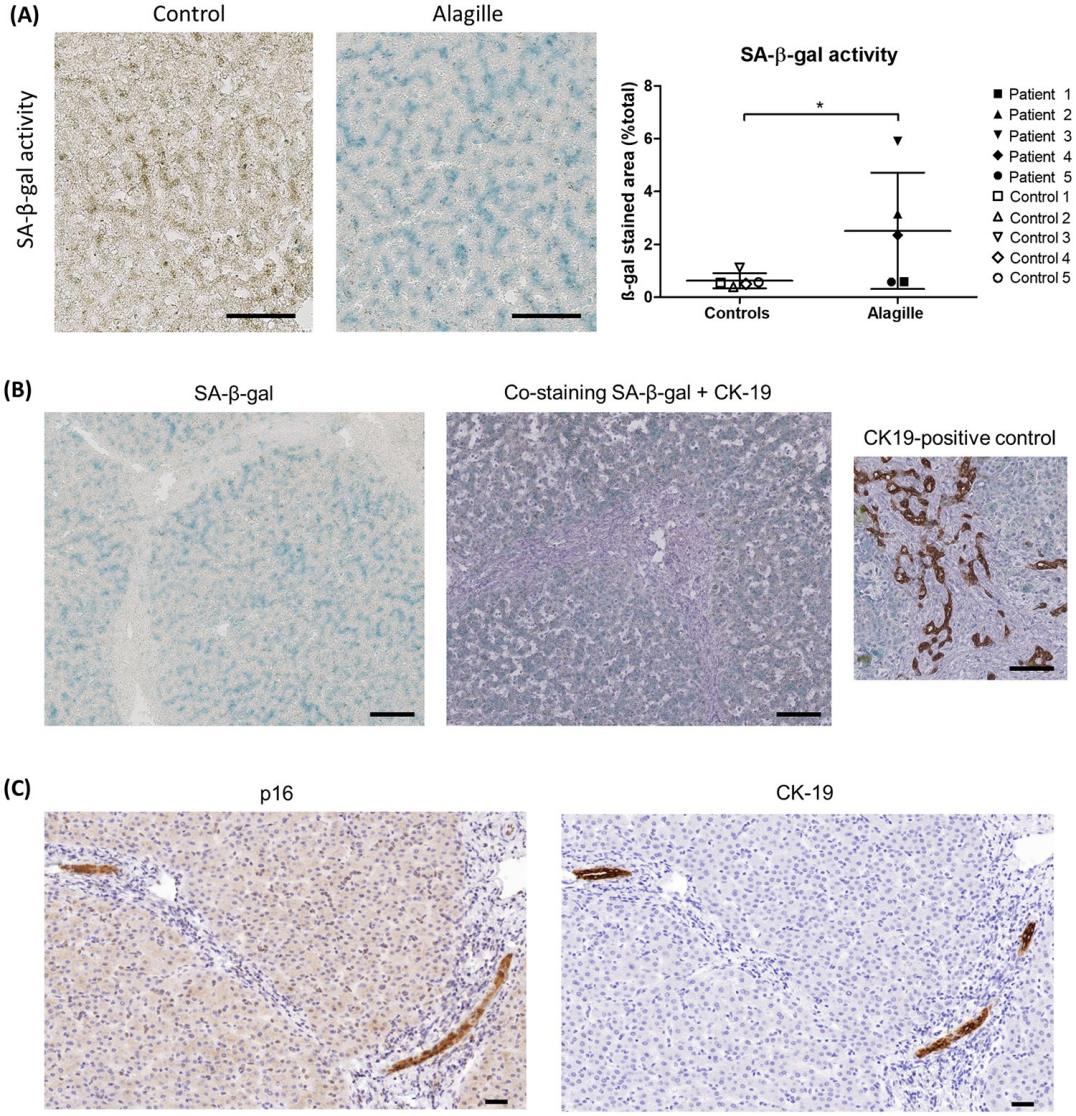
Senescence localization in ALGS livers. (A) SA-β-gal activity increases in ALGS livers as compared to controls. (B) Concomitant staining of SA-β-gal activity and CK-19 IHC shows senescence in the whole liver parenchyma of ALGS livers, with disappearance of intrahepatic bile ducts as demonstrated by the absence of CK-19 immunostaining on this liver section. (C) Serial immunostaining of p16 and CK-19 evidences senescence in the remaining bile ducts of ALGS livers. Liver sections from patient number 2 and control number 2 were used as representative images. ALGS: Alagille syndrome; IHC: immunohistochemistry; SA-β-gal: senescence-associated beta-galactosidase. Data is presented as mean ± SD; *p<0.05. Scale bars = 100 μm.

SA-β-gal activity confirmed the presence of senescence in ALGS livers (Fig 3A). Concomitant staining of SA-β-gal activity and CK-19 immunohistochemistry revealed the presence of senescence in the whole liver parenchyma and pointed out the disappearance of bile ducts in ALGS livers (Fig 3B). However, the remaining bile ducts were senescent as well as demonstrated by serial immunostaining of p16 and CK-19 (Fig 3C). Of note, levels of senescence appeared comparable between adult and pediatric control livers, as expected since cholangiocytes and hepatocytes do not suffer from telomere shortening and replicative senescence until late in life in physiological conditions (Figs 1–3) [10].

Finally, we investigated the expression of senescence and SASP-related genes in a published RNA sequencing dataset from Andersson *et al*, obtained from pediatric ALGS (n = 5) and cholestatic (n = 2)/non-cholestatic (n = 2) control livers (GSE104873) [11]. We only considered the results of the differential expression analysis comparing ALGS to non-cholestatic controls since oxidative stress due to bile acids accumulation can *per se* lead to premature senescence in cholestatic diseases [12]. Also, ALGS and cholestatic livers clustered together in the principal component analysis of the study and were distinct from the transcriptomes of the two non-cholestatic controls suffering from autoimmune hepatitis [11]. Amongst the 370 genes that were upregulated in the differential expression analysis of ALGS livers versus non-cholestatic controls, 14 genes were previously described as SASP components, including *CXCL8* (IL-8) (Table 2) [13–15]. Only 2/5 ALGS patients had a marked upregulation of all the 14 SASP-related genes. *CDKN1A* (p21), *CDKN2A* (p16), *TGFB1* (TGF-β1) and *IL6* (IL-6) were not significantly upregulated in the differential expression analysis, but seemed overexpressed in some ALGS patients when the detailed database was investigated. Those observations underlie the heterogeneity of senescence and SASP-related transcriptomes in ALGS patients.

**Table 2:** SASP-related genes upregulated in ALGS livers versus non-cholestatic controls.

Gene	LogFC	*p*-value
*CXCL8*	7.06	2.99 x 10^−15^
*CCL20*	6.65	3.22 x 10^−11^
*CCL3*	4.04	7.8 x 10^−5^
*PDGFA*	3.75	1.91 x 10^−11^
*IGFBP1*	3.2	1.51 x 10^−7^
*PLAU*	3.12	6.3 x 10^−4^
*LIF*	3.08	1.1 x 10^−3^
*CCL2*	3.07	1.84 x 10^−4^
*IL32*	3.05	9.56 x 10^−7^
*MMP2*	2.96	8.69 x 10^−6^
*PLAUR*	2.7	1.12 x 10^−3^
*TIMP1*	1.9	5.83 x 10^−4^
*ICAM1*	1.87	4.18 x 10^−4^
*MMP14*	1.45	3.09 x 10^−3^

This table was extracted from the differential expression analysis of a published RNA sequencing dataset from Andersson *et al* [11]. SASP genes were identified based on existing literature [13–15]. ALGS: Alagille syndrome; FC: fold change; SASP: senescence-associated secretory phenotype.

## Discussion

Our results demonstrate for the first time that *JAG1* mutated livers from patients with ALGS display advanced premature senescence from early life. Senescence is located in the whole liver parenchyma, and predominates in hepatocytes in the presence of a major intrahepatic ductular paucity inherent to the disease. Our observations are in line with the increased SA-β-gal activity that was evidenced in livers from jag1b/2b mutants zebrafish (jag1b^-/-^; jag2b^-/-^), in which the developmental loss of intrahepatic cholangiocytes and the subsequent cholestasis of ALGS are phenocopied [16]. Due to Jagged1 mutation, an impairment of the juxtacrine transmission of senescence mediated by Jagged1-Notch1 interactions would be expected in ALGS [6]. Jagged1 deficiency might also affect the transient increase in Notch1 and in the active Notch1 intracellular domain (N1ICD) necessary to induce the TGF-β1-rich primary SASP, TGF-β being an important promotor of the paracrine transmission of senescence in liver disease [6, 17, 18]. This transient increase is normally followed by N1ICD downregulation, which allows the production of a pro-inflammatory SASP containing inflammatory cytokines such as IL-6 and IL-8 through the de-repression of C/EBPβ [5]. The SASP markers TGF-β1, IL-6 and IL-8 were not overexpressed in ALGS livers in our cohort–except for one patient–and TGF-β1 was not differentially expressed in ALGS versus control livers in a published RNA sequencing dataset [11]. Jagged1/Notch/TGF-β-independent mechanisms are therefore involved in the important premature senescence that we observe in ALGS livers, in the absence of the classically described mechanisms of juxtacrine and paracrine transmission of senescence. We indeed observed that other SASP components promoting the paracrine transmission of senescence such as CCL2 and CCL20 were upregulated in ALGS livers RNA sequencing dataset, corroborating the fact that other senescence pathways might compensate the abnormal Notch signaling [11, 19, 20]. Also, we observed an important interpatient variability regarding the expression of senescence and SASP-related genes in our cohort and in a published dataset of ALGS livers. The senescence and SASP profiles can vary widely according to the cell type, senescence-inducing stress and duration of senescence, but the interpatient variability that we observed could also be related to the high phenotypic variability inherent to ALGS [15]. Finally, senescence markers were expressed to various extent within one patient, corroborating the fact that the analysis of a combination of markers is mandatory for an accurate evaluation of the senescent phenotype [21]. Further studies will be needed to deeply investigate pathways involved in senescence and SASP regulation, especially due to the small number of patients included in this preliminary study along with high interpatient variability. In particular, comparing *JAG1*- and *NOTCH2*-mutated ALGS livers could be of high interest to discriminate Notch1 and Notch2 involvement in senescence and SASP. In conclusion, ALGS livers display important premature senescence despite Jagged1 mutation, underlying the complexity of senescence and SASP development pathways.

## Supporting information

S1 File Supplementary tables. This file contains the S1 (primary antibodies) and S2 Tables (pre-designed TaqMan probes).
(DOCX)

S2 File Dataset. This file contains the dataset underlying the results described in our manuscript.
(XLSX)
