# Supplementary material for: Premature senescence of the liver in Alagille patients
Source: PLoS One. 2023 Apr 26;18(4):e0285019. doi: 10.1371/journal.pone.0285019 (PMC10132695; doi:10.1371/journal.pone.0285019)
Supplement: S1 File — This file contains the S1 (primary antibodies) and S2 Tables (pre-designed TaqMan probes). (DOCX) [file pone.0285019.s001.docx]

**Supplementary tables**

| **Primary antibody** | **Company** | **Cat. No** | **Species** | **Ag retrieval** | **Dilution** |
| --- | --- | --- | --- | --- | --- |
| p21 WAF1/Cip1 | Agilent | M7202 | M mAb | T 1 hour 98C | IHC 1/400 |
| p16 INK4a | Abcam | ab108349 | Rb mAb | T 1 hour 98C | IHC 1/1000 |
| gamma H2A.X (Ser139) | Abcam | ab81299 | Rb mAb | C 35 min 98C | IHC 1/500 |
| CK19 | Dako | M0888 | M mAb | C 35 min 98C | IHC 1/100 |

**S1 Table.** Primary antibodies. Ag: antigen; C: citrate; M: mouse; mAb: monoclonal antibody; pAb: polyclonal antibody; Rb: rabbit; T: Tris-EDTA.

| **Gene of interest** | **Company** | **Reference** |
| --- | --- | --- |
| *CDKN1A* | Thermo Fisher Scientific | Hs00355782_m1 |
| *CDKN2A* | Thermo Fisher Scientific | Hs00923894_m1 |
| *IL6* | IDT | Hs.PT.58.40226675 |
| *CXCL8* | IDT | Hs.PT.58.38869678.g |
| *TGFB1* | IDT | Hs.PT.58.39813975 |

**S2 Table.** Pre-designed TaqMan probes used for reverse transcription quantitative polymerase chain reaction.
